# Peptide Amphiphiles in Corneal Tissue Engineering

**DOI:** 10.3390/jfb6030687

**Published:** 2015-08-06

**Authors:** Martina Miotto, Ricardo M. Gouveia, Che J. Connon

**Affiliations:** Institute of Genetic Medicine, Newcastle University, International Centre for Life, Central Parkway, Newcastle upon Tyne NE1 3BZ, UK; E-Mails: M.Miotto1@newcastle.ac.uk (M.M.); Ricardo.Gouveia@newcastle.ac.uk (R.M.G.)

**Keywords:** cornea, corneal diseases, peptide amphiphiles, bioactive molecules, tissue engineering, corneal tissue engineering, wound healing

## Abstract

The increasing interest in effort towards creating alternative therapies have led to exciting breakthroughs in the attempt to bio-fabricate and engineer live tissues. This has been particularly evident in the development of new approaches applied to reconstruct corneal tissue. The need for tissue-engineered corneas is largely a response to the shortage of donor tissue and the lack of suitable alternative biological scaffolds preventing the treatment of millions of blind people worldwide. This review is focused on recent developments in corneal tissue engineering, specifically on the use of self-assembling peptide amphiphiles for this purpose. Recently, peptide amphiphiles have generated great interest as therapeutic molecules, both *in vitro* and *in vivo*. Here we introduce this rapidly developing field, and examine innovative applications of peptide amphiphiles to create natural bio-prosthetic corneal tissue *in vitro*. The advantages of peptide amphiphiles over other biomaterials, namely their wide range of functions and applications, versatility, and transferability are also discussed to better understand how these fascinating molecules can help solve current challenges in corneal regeneration.

## 1. Introduction

The cornea is the transparent, outermost part of the eye that serves as the primary refractive organ in the visual system [1]. Diseases, traumas, or injuries are the leading causes of corneal blindness and its prevalence varies from country to country, and even from one population to another, depending on many factors such as availability and general standards of eye care. It is estimated that 180 million people worldwide have severely impaired vision in both eyes, resulting in a considerable social and economic impact [2]. Corneal disease remains a major cause of blindness, second only to cataracts. Although multi-factorial, the vast majority of corneal clinical cases would benefit from a suitable corneal replacement. However, there is currently a lack of donor cornea availability. The main factor behind this donor shortage is that, in many parts of the world, there are limitations in the storage and distribution of corneal tissue, as well as cultural and/or religious barriers [3]. Moreover, the supply of human corneal tissue is expected to diminish even further due to the increasing popularity of refractive surgery (such as LASIK), a technique that renders these corneas unsuitable for donation. However, even considering the best conditions, donor grafts are typically variable in quality and usually fail due to immunological rejection or endothelial decompensation resulting in an 18% failure rate for initial grafts [4].

In the context of these limitations, the field of corneal tissue engineering has made considerable advances in the last 10 years, focusing on alternative means of replacing damaged corneal tissue. Approaches have included development of fully artificial keratoprostheses, use of decellularized tissue scaffolding from animal or human sources, and use of acellular, cross-linked collagen constructs as corneal replacements [5,6]. Presently, bioengineered corneal substitutes are already available for experimental clinical purposes such as corneal grafting [7,8]. In addition to their clinical applications for transplantation and wound healing enhancement, these engineered tissues also represent attractive *in vitro* models of human tissues for various biological purposes. However, whilst much work is going on in this research area [9,10], this review will instead focus on ongoing studies using different biomaterials to create new corneal tissues, and more specifically, work involving peptide amphiphiles (PAs) in corneal tissue engineering. The advantages of using these biomaterials and the significant challenges involved will also be discussed, along with the many future perspectives in the field.

## 2. Challenges in Corneal Tissue Engineering

The final purpose of corneal tissue engineering is the fabrication of corneal tissue equivalents able to improve the function of their injured or diseased natural counterparts. However, constructing a cornea presents several challenges to the field of tissue engineering due to the very specific structural and cellular properties of the organ. Strength, shape, transparency, biocompatibility, and molecular and cellular compositions are important properties of the cornea that remain difficult to replicate *in vitro*. Moreover, assembly and recovery of the engineered corneal tissues whilst maintaining minimal manipulation before and during grafting remains an important part of the bio-fabrication process and still requires intense study and optimization.

**Figure 1:**
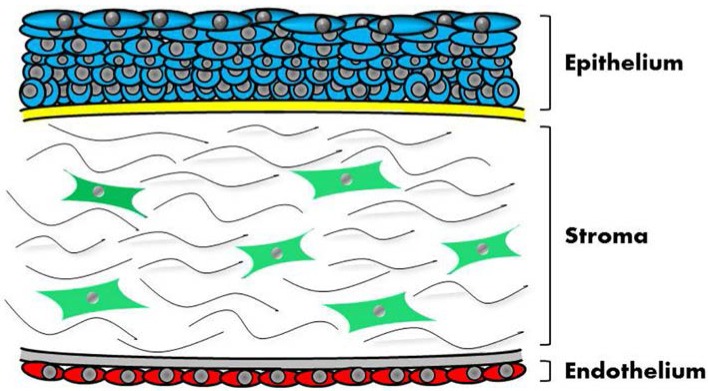
The human central cornea in cross-section. The outer-, anterior-most surface of the cornea comprises a non-keratinized, multi-layered epithelium (*blue*) supported by a basement membrane and above the Bowman’s layer (*yellow*). The middle stromal tissue comprises 90% of the cornea’s thickness, and is sparsely populated with keratocytes (*green*) interspersed within approximately 200 lamellae of dense, collagen- and proteoglycan-rich extracellular matrix (*lines*). The innermost posterior tissue consists of a single layer of endothelial cells (*red*) supported by the Descemet’s membrane (*grey*).

At a macroscopic level, from an anterior to a posterior location, the human cornea is composed of a non-keratinized multi-layered epithelium, the Bowman’s membrane, a 0.5 mm-thick stroma, the Descemet’s membrane, and an endothelium [11] (Figure 1). The stroma accounts for 90% of the volume of the cornea, and is essential to support the mechanical and refractive properties of the organ. These properties are based on the ultrastructural organization of the stroma’s extracellular matrix, comprised by a pseudocrystalline lattice of highly-ordered collagen fibers and proteoglycans, and sparsely populated by quiescent stromal cells, the keratocytes. This arrangement plays a fundamental role in the structure and function of the cornea. Specifically, the orderly array of collagen fibers and the refractive index matching of these fibrils by interstitial proteoglycans play a significant role in the transparency of the tissue [12]. Stromal collagen type-I fibers have a 20–35 nm diameter, and are aligned parallel to each other with regular 30-nm spacing between fibrils. This regular spacing is thought to be regulated by stromal-specific proteoglycans, which have been observed to form ring-like structures around collagen fibrils in the normal cornea [13]. The aligned fibers are grouped into layers called lamellae, which are stacked in an alternating lattice [14]. The thickness of the stroma and arrangement of the collagen fibers are optimal for light transmission through the cornea, with minimal light scatter.

Corneal transparency also has a cellular contribution. Keratocytes express certain proteins, known as corneal crystallins, which are thought to match the refractive index of the cell cytoplasm with the surrounding matrix material [15]. In addition, the cornea is avascular, a property maintained by its anti-angiogenic milieu [16] which, if eliminated, can lead to blood vessel ingrowth and loss of transparency [17]. Moreover, the overall structure of the stroma is dependent on its hydration state, a feature regulated by the corneal epithelial [18] and endothelial layers [19]. Furthermore, the correct development and function of these tissue layers are dependent on the presence of a well-developed network of nociceptive neurons [20]. From this perspective, any attempts to engineer corneal tissues must take into consideration the multi-cellular nature of the cornea, and the intricate direct and indirect interaction maintained between the various tissues.

## 3. Previous Approaches to Engineer Corneal Tissue: Top-Down or Bottom-Up?

In recent years, the development of tissue-engineered corneal substitutes emerged as an alternative method to overcome several issues related to corneal transplantation, namely the relatively high host-rejection rate of keratoprosthesis. In this context, different corneal stromal equivalents have been developed using various biomaterials such as decellularized corneas [21], amniotic membrane [22] or scaffolds produced from collagen type I [23], fibrin agarose [8,24,25,26], fish scales [27], chitosan [28], caprolactone [29], or poly(ester urethane) urea [30]. For instance, Du *et al.* [22] used amniotic membrane as a biomaterial upon which human corneal epithelium stem cells were tested as therapy for limbal stem cell deficiency. Among all the materials used for the production of biocompatible scaffolds, collagen-based constructs seem to be the most interesting. A number of examples can be seen in the work of Griffith and co-workers, where considerable effort was made to create and optimize scaffolds produced from cross-linked collagen [31], recombinant human collagen [32], and bio-functionalized collagen [33] for corneal tissue engineering purposes. Although many of these approaches are currently being tested in the clinic, none has had the broad success and acceptance of fresh tissue transplantation. The reason for this discrepancy might be due to the different strategies used to produce bioengineered tissues in general, and corneal tissue equivalents in particular.

Traditional tissue engineering typically employs what is called a top-down approach. This is based on the use of scaffolds, necessarily biocompatible and optionally biodegradable, to recreate the appropriate microarchitecture of the natural tissue and serve as support for cell attachment and growth. Cells seeded on such materials are expected to populate them while maintaining their native phenotype, and use these scaffolds as support while creating a suitable growth environment (e.g., by depositing their own extracellular matrix). Theoretically, a 3D scaffold with a precise shape, composition and internal organization can provide a perfect microenvironment allowing the organization of individual cells into a functional tissue [34]. However, the design *a priori* of scaffolds with mechanical, physiochemical and biological properties ideal for a specific tissue has not been yet realized, and is probably beyond current knowledge and technology. On the other hand, bottom-up approaches are emerging as an alternative for creating highly organized tissues, and using these modular units as building blocks to engineer biological tissues. These modular units can be fabricated using different methods such as self-assembled aggregation [35], microfabrication of cell-laden hydrogels [36] and extracellular matrix [37], overlapping of cell sheets [38], or direct printing of tissues [39]. Once bio-fabricated, these blocks can be stacked, assembled, or combined to form larger tissues or whole organs [40]. Commonly, the bottom-up approaches aim at providing cells with a guiding template to direct cell-driven organization and tissue formation. In other words, cells are instructed to recapitulate natural tissue differentiation, growth and morphogenesis *in vitro*. These strategies have allowed the creation of modular tissues with native-type composition and micro-architecture, without the need to introduce scaffolds and with better perceived outcomes in downstream applications (e.g., grafting). The difference between top-down and bottom-up strategies constitutes an important topic for future approaches to corneal tissue engineering, as discussed in a recent review article focused on the subject [41].

## 4. Peptide Amphiphiles in Tissue Engineering

**Figure 2:**
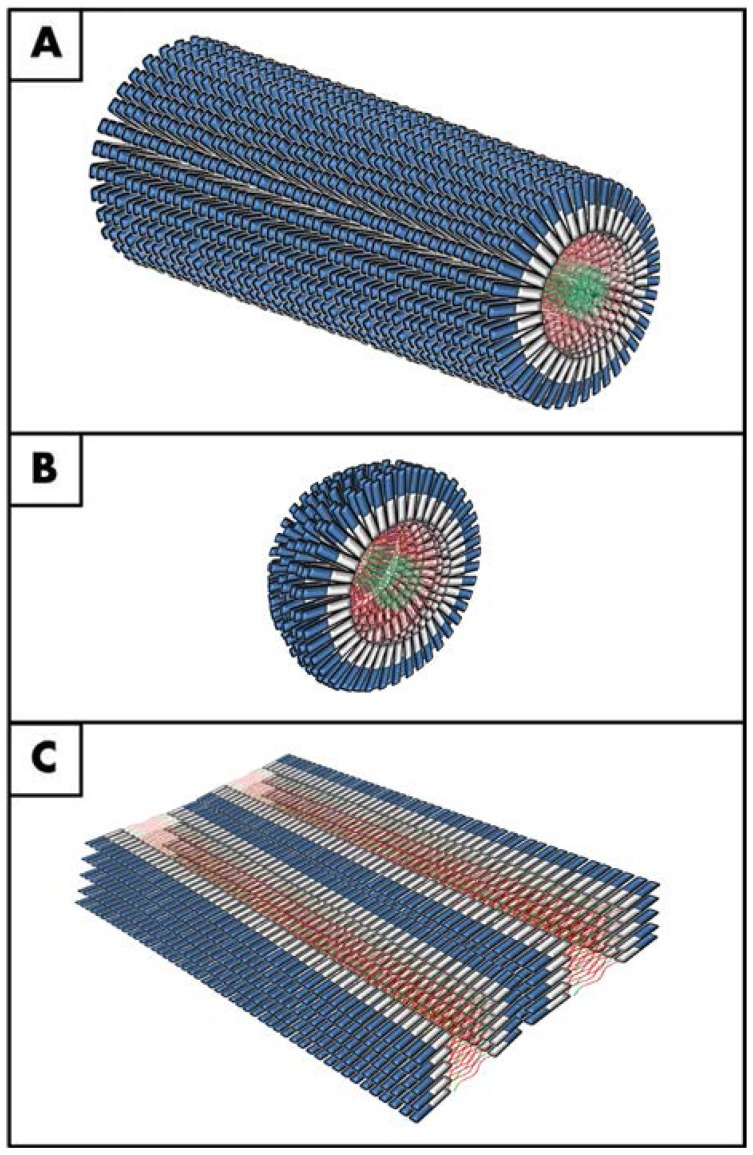
Examples of supramolecular PA nanostructures. Graphical representation of some representative structures obtained by PAs self-assembly: (**A**) nanofibers; (**B**) micelles; and (**C**) multi-layered nanotapes. All three structures have a hydrophilic outer corona comprised of bioactive peptide (*blue*) and self-assembly-inducing/spacer sequence (*white*), and a hydrophobic inner core with organized and/or non-organized PA tails (*red* and *green*, respectively) (*adapted from* [63,79]).

Recently, small bioactive molecules capable of self-assembly have attracted considerable interest as new functional materials with broad applications in tissue engineering and regenerative medicine [42,43,44]. Specifically, these are self-assembling molecules used to produce biocompatible materials for three dimensional cell culture [45,46], drug delivery [47,48], inhibition of bacterial growth [49,50], delivery of therapeutic molecules [51], or as scaffolds for cell therapy [52,53,54,55]. Concerning their use as delivery systems, it is important to understand if and how supramolecular nanostructures can cross the diffusion barriers present in the human body such as the blood-brain barrier or, relevant to corneal applications, the corneal epithelial or endothelial layers. One of the most promising types of such molecules comprises small synthetic peptides. These molecules incorporate small bioactive or bio-inspired peptide sequences, with several advantages over the use of whole-protein matrixes, including sourcing (*i.e*., easier isolation/production and purification), reduced immunogenicity [56], presentation (*i.e.*, more effective and controlled density and orientation of the bioactive motives [57]), and stability [58]. In addition, they can be rationally designed to have amphiphilic characteristics, *i.e.*, to contain both hydrophilic and hydrophobic domains that help them self-assemble into a variety of supramolecular 3D nanostructures, such as tubes, tapes, fibres, vesicles and micelles, among other architectures [52,59,60,61] (Figure 2). However, despite this variability, or maybe because of it, there are currently no set rules for this rational design. In other words, there is still much work to be done regarding the development of a supramolecular code that will allow us to predict the self-assembly of hierarchical architectures and bio-function based solely on the primary structure of amphiphilic peptides [62].

Amphiphilic peptides can be classified as peptide sequences with amphiphilic properties arising from hydrophobic and hydrophilic residues, whereas peptide amphiphiles (PAs) constitute a subset of the former comprising a peptide sequence linked to a hydrophobic tail [63]. PAs can be easily synthesized by standard peptide synthesis protocols by standard solid phase chemistry that ends with the alkylation of the NH_2_ terminus of the peptide; their structural folding and stability have been extensively characterized [64,65]. However, and although small and medium-sized peptides are easily obtained in high yields, large peptide sequences (*i.e*., longer than 50 amino acids) are still difficult to produce and purify by direct chemical synthesis [66]. An example of a representative PA contains three segments: a hydrophobic sequence, commonly a lipid chain that guides aggregation through hydrophobic collapse, a β-sheet-forming peptide that promotes nanofiber formation through the formation of hydrogen bonds, and a peptide segment, usually less than 15 amino acids long, with ionisable side chains and a bio-functional amino acid sequence [67]. In this review we will give specific attention to the characteristics and applications of such PAs.

The self-assembly mechanism involved in these single-tailed PAs usually occurs after changes in pH [68], mixing of oppositely charged PAs [69], or addition of multivalent cations [54] to generate electrostatic repulsion between the PA molecules. The supramolecular self-assembly of PAs in aqueous environments is governed by at least three major forces: the interactions between the hydrophobic tails, the electrostatic repulsions between charged groups, and the hydrogen bonding among the middle peptide segments [52]. The derived ultrastructure of self-assembled PAs reflects a balance of each force contribution, and has dimensions similar to those of fibrils from natural extracellular matrix. Specifically, taking advantage of their amphiphilic properties, the hydrophobic alkyl tails of PAs solubilized in aqueous solutions are packed in the center of the fiber while the hydrophilic peptide segments are exposed to the aqueous environment, forming an external corona. As such, these molecules can be designed to display bioactive epitopes at the surface of the self-assembled nanostructure, while keeping intermolecular hydrogen bonds parallel to the long axis of the fiber [52]. To date, a considerable number of PAs have been reported in the literature [70], including molecules with different hydrophobic tails [71,72,73]. For example, PAs comprised of a similar peptide sequence but with either saturated or diene-containing hexadecyl lipid chains self-assemble into polydisperse nanotapes or spherical micelles, respectively [44,74]. These examples illustrate the versatility of PAs, where increasing unsaturation and length of the lipid chains, or changing from alkyl to aromatic tails dramatically alters the final architecture of the self-assembled nanostructures [75]. However, this feature might compromise the stability, physical properties, and function of the PA, namely when exposed to UV [76] and γ-irradiation [77], two common sterilization methods used in materials for biological applications. Moreover, the concentration in which these molecules are used may constitute an important factor defining their self-assembled architecture [78] and bio-compatibility [42].

Some of the main applications of PAs in regenerative medicine are summarized in Table 1. As reported in Table 1, PAs can be used in different forms, such as in coatings, in solution, or as hydrogels. All of these forms have been quite extensively tested, however, in spite of the advantages of using PAs in hydrogel form, it has to be considered that they exhibit poor mechanical characteristics [80]. In this context, the use of PAs as hydrogels is less suited to the production of scaffolds for engineering tissues requiring high mechanical strength and integrity. Considering the application of PAs in the field of tissue engineering, these molecules have been used by several groups towards the development of engineered constructs, particularly for the regeneration of connective tissues with a collagen-rich extracellular matrix. In order to achieve this objective using scaffold-based approaches, an artificial PA scaffold should mimic the structure and biological function of the native extracellular matrix as much as possible, both in terms of chemical cues and physical and mechanical properties. The native extracellular matrix provides structural support to body tissues, acting not only as a physical framework for arranging cells within the connective tissues, but also as a dynamic and flexible substance defining cellular behaviour and tissue function [81]. Indeed, it has been shown that the supramolecular network formed by self-assembled PA mimics, from a structural point of view, the natural extracellular matrix, albeit in a simplified way. In this context, the main applications of PAs molecules, so far, have been in the repair of bone [82,83], cartilage and tendon [84], blood vessels [85,86], cavernous nerves [87], skin [88,89], and importantly for this review, the cornea [44,74].

## 5. Peptide Amphiphiles as Versatile Templates to Recreate Human Corneas *in vitro*

Various biomaterials have been explored for use in tissue engineered corneal substitutes, including, but by no means limited to, collagen [23,90,91], fibrin-agarose [8], decellularised cornea [92], and amniotic membrane [93,94]. Considering the high transparency of the corneal tissue, it is fundamental to design a scaffold preserving this characteristic whilst maintaining a high biocompatibility and a low immunogenicity [95]. In addition, there is a strong need to develop novel bioactive materials able to support cell adhesion and proliferation. In this context, PAs are well placed in that they can be designed to support a range of cell types important to corneal function, *i.e.* keratocytes, epithelial and endothelial cells. In this case the use of PAs would not only support the formation of extracellular matrix-inspired nanofibers following the self-assembly, but also enhance adhesion, proliferation, and alignment of human corneal stromal fibroblasts due to the insertion of specific bioactive motives. Notable research involving PAs in cornea tissue engineering are reported in Table 2.

**Table 1:** Main works involving the use of peptide amphiphiles in regenerative medicine. The table reports the PA used, its chemical structure, the aim of the studies, the source of the bioactive sequence, the concentration of PA used, and its form.

PA	Chemical Structure	Aim	Source	[PA] wt %	PA Form	Reference
C_16_-C_4_-G_3_-S-RGD	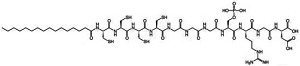	Bone regeneration	Fibronectin	0.1	coating	[83]
C_12_-HSNGLPLGGGS EEEAAAVVV(K)	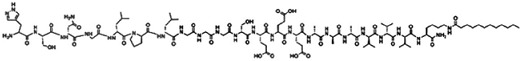	Cartilage regeneration	*De novo* synthetized	1	hydrogel	[84]
C_16_-V_2_A_2_E_2_-NH_2_	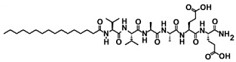	Cavernous nerve regeneration	*De novo* synthetized	0.85	hydrogel	[87]
C_16_-C_4_-G_3_- LRKKLGKA	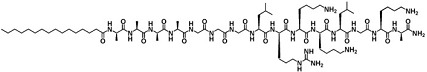	Blood vessels regeneration	Heparin binding consensus sequence	3	hydrogel	[85]
C_16_-KTTKS	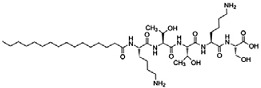	Skin regeneration	Procollagen I	0.0003	solution	[88]

**Table 2:** Main works involving the use of peptide amphiphiles in corneal tissue engineering. The table reports the PA used, the source of the bioactive sequence, the PA biological effect, the concentration of PA used, and its form.

PA	Source	Biological Effect	[PA] wt %	PA Form	Reference
C_16_-G_3_-RGD/RGDS + C_16_-ETTES	Fibronectin	Enhanced adhesion and proliferation of hCSFs	1 to 0.005	coating	[42,96]
A_6_-RGDS	Fibronectin	Enhanced adhesion and proliferation of hCSFs	1 to 0.1	coating	[97]
C16-TPGPQGIAGQ-RGDS	MMP cleavage sequence + Fibronectin	Promoted adhesion andgrowth of hCSFs. Stimulated collagen production. Governed tissues lift-up	2	coating	[44,74]
Fmoc-RGDS	Fibronectin	Enhanced cell attachment, proliferation and viability	1	solution	[43]
C_16_-KTTKS	Procollagen I	Stimulated collagen production from hCSFs	0.002, 0.004, 0.008	solution	[98,99]
C_12_-VVAGKYIGSR	Laminin	Enhanced keratocyte proliferation and migration, and stimulated collagen I synthesis	0.2	coating	[100]
C_16_-YEALRVANEVTLN	Lumican	Stimulated collagen I production	0.01, 0.005, 0.0025 0.00125, 0.000625	solution	[101]

For corneal tissue engineering one of the most used bioactive sequences is represented by the fibronectin derived RGD motive. As extensively reported, this sequence is frequently used for creating adhesive biomaterials able to promote cell interaction and adhesion through binding to integrin subgroups like those composed of subunits αV, α3β1, and α5β1 [102,103]. However, even if the tripeptide motif RGD has been identified as a minimal essential cell adhesion sequence [104], the definition of the *minimo optimo* bioactive epitope required in order to have the same function as the whole protein, is still not known. In this respect, Castelletto *et al.* [96] studied the self-assembly of two PAs designed *ad hoc* to enhance the potential for cell attachment. These peptides contained RGD or RGDS bioactive motives as well as a functional spacer in the β-sheet domain, consisting of a sequence of three consecutive glycine (G) residues. In addition, they optimized the potential for cell attachment by co-assembling the bioactive PA (RGD or RGDS) with a non-bioactive PA acting as a diluent molecule able to vary the RGD density within its supramolecular form following self-assembly above critical aggregation concentration. In particular, they used a negatively charged PA composed of C_16_-Glu-Thr-Thr-Glu-Ser (C_16_-ETTES) as this diluent. Castelletto *et al.* posited that by tuning the diluent concentration, so the distance between neighbouring RGD groups would be greater than that in the undiluted RGD(S)-PA following self-assembly, they could optimize the PA for maximal cell attachment. When such a PA mixture was subsequently used as a coating for 2D human keratocyte attachment and growth, the optimal molar ratio of C_16_-RGD(S):C_16_-ETTES was found to be 15:85. Subsequently, this approach was employed by Gouveia *et al.* [42] to produce stable biocompatible film coatings which enhanced adhesion, proliferation and alignment of human corneal stromal fibroblasts whilst inducing the formation of 3D lamellar-like stromal tissue. These early results suggested that such mixtures of PAs constitute a promising new material capable of directing corneal stromal cells to produce appropriate amounts and type of extracellular matrix that are likely to be important to both corneal wound healing and tissue engineering. However, the biophysical, mechanical, and biological properties of these functional coatings require further extensive research. Castelletto *et al.* [97] also demonstrated the ability to produce bioactive film coatings for corneal stromal cell growth using an alanine-rich amphiphilic peptide containing the RGD motive (A_6_-RGD). This PA was designed to simultaneously ensure solubility in water and specific binding to cells. They found that the self-assembly motive depended on the concentration of surfactant-like peptide (SLP) and demonstrated the co-existence of vesicles and fibres with an increase in vesicle population relative to fibres when the SLP concentration increases. Moreover, at low concentrations (0.1 wt % –1.0 wt %), this SLP promoted adhesion and enhanced proliferation of human corneal stromal cells. Thus, A_6_-RGD PA represents another promising bioactive peptide for the manufacture of dry coatings for cornea tissue engineering.

**Figure 3:**
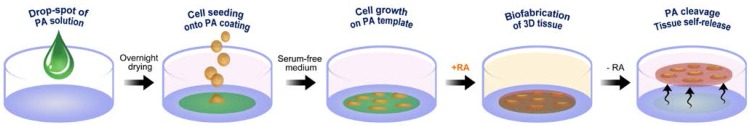
Bio-fabrication and controlled self-release of live tissues using PA coating templates. Schematic representation of the method used for the *in vitro* bio-fabrication and lift-off of human corneal stromal tissues, previously reported in [74]. Cells isolated from human donors were seeded and grown on low-attachment plates previously coated with a PA carrying both the MMP1-sensitive sequence and the RGDS cell adhesion motive. Cells were cultured in serum-free medium containing retinoic acid (RA) for 90 days and accumulated large quantities of corneal-specific stromal extracellular matrix. Subsequently, the bio-fabricated tissues were induced to express MMPs due to RA removal from the medium. In three days, the tissues expressed enough endogenous MMPs into the culture supernatant to provide the cue to degrade the adhesive PA coating, and induce their own release. The resulting free-floating corneal stromal equivalents were scaffold-free, easy to handle, and retained their shape and structural integrity for more than 18 months in storage.

More recently, a study by Gouveia *et al.* [74] described an inventive way to take advantage of PA film coatings for corneal tissue engineering. In this work, they described a biologically interactive PA coating that integrated both the abilities to induce tissue bio-fabrication and the subsequent tissue self-release *in vitro*. This was made achievable by employing a custom designed PA comprised a matrix metalloprotease (MMP)-cleavable sequence (Thr-Pro-Gly-Pro-Gln-Gly-Ile-Ala-Gly-Gln) followed by the RGDS bioactive motive which, as before, was mixed with C_16_-ETTES at a molar ratio of 15:85. The subsequent self-assembly studies revealed that the PA self-assembled into nanotapes that were also capable of forming film coatings providing a stable surface for the attachment and growth of human corneal stromal cells in a quiescent phenotype. Furthermore the supplementation of all-*trans* retinoic acid (RA) to the culture media facilitated both retained cellular attachment and ultimately tissue formation via cell stratification. Previously Gouveia and Connon [105] had shown that the addition of RA inhibits the expression of several MMPs from the cells while enhancing their native extracellular matrix production. Thus, in the presence of the MMP-sensitive PA coating, the cells increased MMP expression and endogenous proteolytic activity following RA removal. The resulting increase in proteolytic activity in the culture supernatant cleaved the RGD peptide from the self-assembled PA (*i.e*., the nanotape structures underpinning the tissue growth) facilitating the complete detachment of the tissue from the bioactive surface, and creating a free-floating construct (Figure 3). As such, this smart PA material represents a new and fascinating method for the bio-fabrication of certain structural tissues including corneal stromal equivalents.

**Figure 4:**
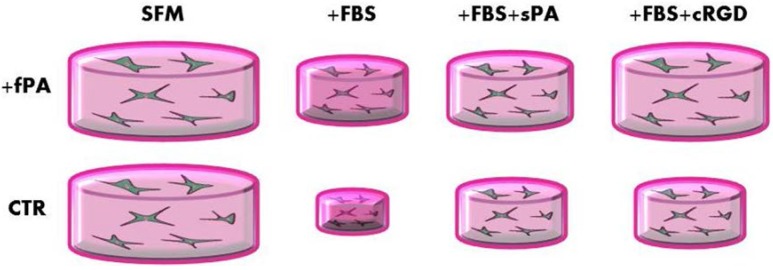
Schematic representation of the effect of Fmoc-RGDS PA on collagen gel contraction under different culture conditions. Human corneal stromal fibroblasts were encapsulated within uncompressed collagen gels that have been functionalized with the fibril-forming Fmoc-RGDS PA (+fPA), or produced without it (CTR). The relative contraction of collagen gels after seven days in serum-free medium (SFM) was negligible, but significantly minimized by the presence of the structural PA in serum-containing media alone (*+*FBS) or supplemented with 50 µM of soluble PA (*+*sPA) or cyclic RGD peptide (*+*cRGD) (adapted from [43]).

PAs have not only been shown to augment the amounts of extracellular matrix produced by corneal stromal cells. For instance, PAs were also used to successfully control the form and shape of corneal tissue-engineered constructs. It is known that during embryonic development, corneal fibroblasts play a central role in exerting physical forces that organize the extracellular matrix into a unique pattern providing structural support whilst maintaining tissue transparency [106,107,108,109,110]. Furthermore, corneal fibroblasts guide wound contraction and matrix remodeling after injury or refractive surgery [14,111,112]. In the physiological process of corneal wound healing, quiescent corneal keratocytes switch to the fibroblast phenotype and migrate through the tridimensional matrix to restore it. In order to investigate cell-matrix mechanical interactions during fibroblast migration, Petroll *et al.* [113,114] developed a model in which cell-seeded compressed collagen matrices are nested within acellular uncompressed matrices. They found that matrices cultured in medium containing exogenous serum become significantly deformed due to keratocytes’ activation and migration into the outer matrix [115]. Similar levels of matrix contraction can disrupt or damage the unique and functional architecture of the corneal stroma leading to the formation of scars or fibrosis. Therefore, a method able to limit it or to stimulate a low contractility migration is highly required. In this respect, a new approach for the containment of the collagen contraction by corneal fibroblasts has been developed through the novel use of PAs. In this example, again by Gouveia *et al.* [43], hybrid materials were constructed comprising fibrillar collagen type I and a PA formed from Fmoc-RGDS. Once encapsulated within this mixture, and exposed to serum, human keratocytes were unable to contract the collagen gel as they would normally (Figure 4). It is believed that this hybrid collagen PA system forms an interpenetrating “gel within a gel” and that the cells preferential bind to the Fmoc-RGDS motif inhibiting the cells from binding to and contracting the collagen gel in the presence of serum. Thus the degree of tissue contraction and shape was controlled by the concentration of the Fmoc-RGDS PA within the system. This interesting study opens up the possibility of using PAs to control localized effects on cells in a three-dimensional environment, thereby lifting the veil on much more complicated tissue-engineered constructs rather than the homogenous forms that are prevalent today.

Another PA showing promising application in corneal wound repair is represented by C_16_-KTTKS. This particular peptide is used in anti-wrinkle cosmeceutical applications under the trade name of Matrixyl. In the first published study on the effects of this commercially available PA, Castelletto *et al.* [98] investigated the self-assembly of this PA in aqueous solution, observing tape-like nanostructures with a broad distribution of widths and reporting that the internal structure of these nanotapes comprised PA bilayers. Subsequently, the work of Jones *et al.* [99] showed that this peptide was able to stimulate collagen production from human corneal stromal cells (as well as dermal fibroblasts) in a concentration-dependent manner. This finding paved the way for a more recent investigation using another potent extracellular matrix stimulatory peptide sequence in the form of a PA. In this case the bioactivity of a PA presenting a lumican-derived bioactive sequence (C_16_-YEALRVANEVTLN) was studied [101]. Lumican is a proteoglycan playing a structural role through binding to fibrillar collagens and modulating fibril formation whilst regulating interfibrillar spacing. The choice of this specific sequence is due to the fact that previous studies have shown it to have matrikine properties [116] such as creating chemokine gradients [117] and promoting the healing of corneal epithelial wounds [118]. Interestingly, this PA self-assembled in twisting and curving tape-like structures, on the borderline between nanotape and fibril structures. Regarding its functionality, this PA has been shown to stimulate collagen production in a concentration-dependent manner, indicating its potential use in tissue engineering and bio-fabrication. A further highly-represented protein family in the extracellular matrix is that of laminins. It is well known that laminins trigger and control many cellular functions [119], rendering them suitable candidates for the design of new bioactive PAs. In this respect, Uzunalli *et al.* [100] reported an interesting study regarding the application of a PA carrying a laminin-derived sequence for corneal stroma regeneration. In particular, this peptide contained the sequence YIGSR derived from laminin β-chain, which regulates cell adhesion through binding to the laminin binding protein (LBP). Their results demonstrated that the PA containing the laminin-derived sequence enhanced cell proliferation, keratocyte migration, and collagen I synthesis, both from *in vitro* and *in vivo*, and to a greater extent compared to the more commonly used RGD-PA. For this reason, this specific bioactive sequence should be considered (alongside the others previously mentioned) in future studies involving corneal stroma bio-fabrication and regeneration.

## 6. Future Perspectives

It can be argued that PAs are “eclectic” molecules, a notion related to the fact that: (1) these molecules can be used in different forms (e.g., as colloidal solutions or hydrogels, as well as thin coating films); (2) PAs can self-assemble in distinct supramolecular structures depending on the molecule’s chemical structure; (3) PAs can have a wide range of biological activities depending on the type and number of bio-functional motives incorporated; (4) PAs can be easily designed and used by researchers with limited expertise in synthetic and organic chemistry; and (5) PAs can be used for various and specific applications in multiple fields, including biochemistry, stem cell biology, biotechnology, and regenerative medicine. Upon evaluating the state-of-the-art on the application of PAs in corneal tissue engineering, it is evident that intriguing and promising results have been achieved recently. In this regard, the potential next step would involve studies concerning the response and the mechanism of integration of these constructs in animal models, eventually followed by clinical trials. From this review, the possibility of abandoning scaffolds (*i.e*., the top-down approach) also emerges . Indeed, it has been shown that the bio-fabrication of corneal stromal tissue can be achieved entirely by human keratocytes instructed by a smart, multi-functional PA coating template. Despite the challenges involved in this work, current research constitutes the foundation and inspiration for the bio-fabrication of other corneal tissues. With such a premise, it is reasonable to conduct further studies in order to further produce multi-cellular, multi-layered, whole-thickness corneas *in vitro*. Moreover, the production of such artificial constructs paves the way for a wider range of applications. For instance, bio-fabricated corneal constructs could be used in pharmacological studies as models to test the effect of new drugs rather than using animals. Indeed, the development of alternative methods that avoid or significantly reduce the use of animals for scientific purposes is still an important issue. In addition, PA templates could be designed to bio-fabricate both corneal and other connective tissues using other cell sources, such as mesenchymal or induced pluripotent stem cells, instead of adult cells. This strategy would be useful for creating multi-cellular constructs in one step, possibly through the use of patterned, multi-component PA systems with numerous specific bioactive peptide motives designed to affect individual cell types. Moreover, since the native ECM proteins are exceptionally multifunctional while PAs typically only possess one bioactive sequence, it would be extremely interesting to design and extensively test the self-assembling and function of PAs carrying more than one epitope, or combine different bioactive PAs molecules in the same system.

## 7. Conclusions

The range of applications for PA-based nanomaterials is steadily expanding, with emerging uses as modulators of extracellular matrix production, and as templates of increasingly complex structure and composition. This wide range of purposes is rooted in the fact that PAs can be rationally designed, and where the primary, secondary, and tertiary structures of PAs depend on the *a priori* aim of the study. Moreover, the use of PAs is continuously evolving, with new discoveries on the function of specific peptide motives and advances in understanding supramolecular chemistry facilitating the development of novel molecules. Although there are several studies involving the use of PAs in biological systems, few are focusing on their application in corneal tissue engineering and repair. This review has highlighted this nascent but potentially useful specialized field, and demonstrates what can be achieved from interdisciplinary collaboration among researchers from the physical, chemical, biological, and clinical sciences. In this context, the current PA studies represent an important step in the change of paradigm for tissue engineering and regenerative medicine.
